# The Next Frontiers in Stroke Prevention: Optimizing Left Atrial Appendage Closure in Atrial Fibrillation: Current Devices, Imaging-Guided Strategies, and Emerging Approaches

**DOI:** 10.3390/jcm15176526

**Published:** 2026-08-24

**Authors:** Zain Al-Abdeen Mohammed Qassim, Mohamedanas Mohamedfaruk Patni, Biji Thomas George, Subhranshu Sekhar Kar, Rajani Dube, Reema Mohammed Saeed Al Shaibani, Ghina Hasan Yassin, Yara Mamoun Theyabat, Ibrahim Alabid, Malak Mohammed Qassim

**Affiliations:** 1 RAK College of Medical Sciences, Ras Al Khaimah Medical and Health Sciences University (RAKMHSU), Ras Al Khaimah 11172, United Arab Emirates; reema.24901040@rakmhsu.ac.ae (R.M.S.A.S.); ghina.24901033@rakmhsu.ac.ae (G.H.Y.); yara.24901030@rakmhsu.ac.ae (Y.M.T.); ibrahim.21901097@rakmhsu.ac.ae (I.A.); malak.21901022@rakmhsu.ac.ae (M.M.Q.); 2 Department of Community Medicine, RAK College of Medical Sciences, Ras Al Khaimah Medical and Health Sciences University (RAKMHSU), Ras Al Khaimah 11172, United Arab Emirates; mohamedanas@rakmhsu.ac.ae; 3 Department of Surgery, RAK College of Medical Sciences, Ras Al Khaimah Medical and Health Sciences University (RAKMHSU), Ras Al Khaimah 11172, United Arab Emirates; 4 Department of Paediatrics, RAK College of Medical Sciences, Ras Al Khaimah Medical and Health Sciences University (RAKMHSU), Ras Al Khaimah 11172, United Arab Emirates; subhranshu.kar@rakmhsu.ac.ae; 5 Department of Obstetrics and Gynaecology, RAK College of Medical Sciences, Ras Al Khaimah Medical and Health Sciences University (RAKMHSU), Ras Al Khaimah 11172, United Arab Emirates; rajani.dube@rakmhsu.ac.ae

**Keywords:** atrial fibrillation, left atrial appendage closure, stroke prevention, oral anticoagulation, direct oral anticoagulants, watchman, Amplatzer Amulet, cardiac computed tomography, device-related thrombus, peri-device leak

## Abstract

**Background/Objectives**: Atrial fibrillation (AF) is the most common sustained cardiac arrhythmia and is associated with ischemic stroke, systemic thromboembolism, heart failure, and mortality. Although oral anticoagulation (OAC) remains the cornerstone of stroke prevention, long-term therapy may be limited by bleeding risk, contraindications, intolerance, nonadherence, or thromboembolic events despite treatment. Left atrial appendage closure (LAAC) has therefore emerged as a non-pharmacological alternative for selected patients with nonvalvular AF. This review aimed to synthesize current evidence on LAAC, focusing on comparative efficacy versus OAC, device evolution, imaging-guided strategies, antithrombotic management, procedural optimization, unresolved challenges, and emerging technologies. **Methods**: This state-of-the-art narrative review was informed by targeted, non-systematic searches of PubMed and Scopus for publications from January 2009 to June 2026. Evidence was selected purposively to represent clinically relevant guidelines, randomized trials, registries, comparative observational studies, systematic reviews, meta-analyses, imaging studies, device-comparison studies, and reports of emerging technologies. The selected evidence was synthesized narratively; no exhaustive screening process, PRISMA flow diagram, formal risk-of-bias assessment, or quantitative synthesis was performed. **Results**: Randomized evidence was context dependent. OPTION supported LAAC as an alternative to oral anticoagulation in selected anticoagulation-eligible patients undergoing atrial fibrillation ablation. CHAMPION-AF established noninferiority of Watchman FLX to non–vitamin K antagonist oral anticoagulants for its composite efficacy endpoint in a predominantly moderate-risk, anticoagulation-eligible population and demonstrated less non-procedural bleeding, although ischemic stroke occurred numerically more often after LAAC. In contrast, CLOSURE-AF did not establish noninferiority of LAAC to physician-directed medical therapy in an older population at high risks of both stroke and bleeding. These findings indicate that the comparative value of LAAC depends strongly on patient selection, comparator therapy, endpoint composition, procedural risk, and trial design. Observational, registry, and emerging evidence was considered supportive or hypothesis-generating rather than equivalent to randomized evidence. OPTION enrolled only patients undergoing or recently undergoing AF ablation who were suitable for anticoagulation. CHAMPION-AF included 3000 anticoagulation-eligible patients but had relatively low baseline bleeding risk, while CLOSURE-AF studied 912 substantially older, higher-risk patients and did not meet its noninferiority criterion. **Conclusions**: LAAC has evolved into a precision-guided intervention integrating patient risk, left atrial appendage anatomy, device choice, imaging, antithrombotic therapy, and structured surveillance. Further studies are needed to optimize patient selection, standardize post-procedural therapy, and clarify its long-term role compared with contemporary direct oral anticoagulant therapy.

## 1. Introduction

Atrial fibrillation (AF) is the most common sustained cardiac arrhythmia worldwide and remains a major contributor to ischemic stroke, systemic thromboembolism, heart failure, and cardiovascular mortality. AF increases the risk of ischemic stroke by nearly fivefold, and AF-related strokes are often associated with greater disability, higher mortality, and increased healthcare utilization than strokes of other etiologies. As life expectancy rises and cardiovascular risk factors become more prevalent, the global burden of AF is expected to increase, reinforcing the need for effective and durable stroke-prevention strategies [1,2].

Oral anticoagulation (OAC) remains the recommended first-line therapy for most patients with AF who are at elevated thromboembolic risk. Vitamin K antagonists (VKAs) and direct oral anticoagulants (DOACs) substantially reduce ischemic stroke and systemic embolism. However, long-term anticoagulation may be limited by major bleeding, renal dysfunction, drug interactions, frailty, poor adherence, medication intolerance, and absolute or relative contraindications. Some individuals also remain at risk of thromboembolic events despite appropriate therapy, creating a need for alternative strategies that reduce stroke risk while minimizing the limitations of lifelong systemic anticoagulation [1,2,3].

Recognition of the left atrial appendage (LAA) as a predominant site of thrombus formation in nonvalvular AF has transformed contemporary stroke prevention. Although the frequently cited estimate that more than 90% of cardiac thrombi originate in the LAA derives largely from selected historical and surgical populations, the strong association between the LAA and thromboembolism provides the mechanistic rationale for appendage exclusion. LAAC has therefore emerged as an alternative stroke-prevention strategy for selected patients with contraindications to long-term anticoagulation, poor anticoagulant tolerance, or an unfavorable individualized risk–benefit profile. Patient preference represents a separate consideration within shared decision-making rather than an equivalent to an absolute contraindication to anticoagulation [1,2,4,5].

The field has now progressed beyond simple mechanical occlusion. Advances in device engineering, multimodality imaging, procedural technique, and post-procedural management have shifted LAAC toward a precision-guided structural intervention. Contemporary practice increasingly emphasizes individualized device selection, anatomy-guided planning, complete sealing as a procedural goal, tailored antithrombotic therapy, and structured surveillance. Optimizing outcomes, therefore, depends not only on successful implantation but also on integrating patient-specific anatomical, procedural, and clinical factors throughout the entire treatment pathway [3,5,6,7].

This state-of-the-art narrative review examines the contemporary landscape of LAAC in AF, including evidence supporting its use, current and emerging devices, imaging-guided strategies, procedural optimization, unresolved challenges, and future directions. By integrating recent evidence with evolving clinical practice, it highlights the transition of LAAC from a mechanical occlusion procedure to a precision-guided strategy for individualized stroke prevention.

Rather than providing another device-centered overview, this review uses a clinically oriented framework to address five practical questions: which patients should currently be considered for LAAC; how device and procedural strategy should be selected; how imaging should be used before, during, and after the procedure; what post-procedural antithrombotic strategy is supported by current evidence; and which emerging approaches are sufficiently mature to influence contemporary practice. Its distinctive focus is therefore the integration of patient selection, anatomy, device choice, procedural strategy, imaging, antithrombotic management, surveillance, and evidence maturity across the longitudinal LAAC pathway. In this review, LAAC is used as the umbrella term for appendage-closure strategies, whereas LAAO is retained when it forms part of an official study, guideline, or source title.

## 2. Narrative Literature Search and Evidence Selection

This state-of-the-art narrative review was designed to provide a clinically oriented synthesis of contemporary evidence on left atrial appendage closure (LAAC) in atrial fibrillation, with emphasis on patient selection, comparative efficacy versus oral anticoagulation, device evolution, multimodality imaging, post-procedural antithrombotic management, procedural optimization, unresolved challenges, and emerging technologies. To inform the review, targeted searches of PubMed and Scopus were conducted for publications from January 2009 to June 2026. Searches were last updated on 30 June 2026, and the review was limited to English-language publications. Because this was a narrative rather than a systematic review, formal duplicate-removal procedures and study-by-study exclusion-reason logging were not undertaken. Recent evidence was emphasized when methodologically informative, while older landmark studies were retained when necessary to provide historical and clinical context.

The search combined “atrial fibrillation” with “left atrial appendage closure” or “left atrial appendage occlusion”, together with terms related to “Watchman”, “Amplatzer Amulet”, “oral anticoagulation”, “direct oral anticoagulants”, “peri-device leak”, “device-related thrombus”, “cardiac computed tomography”, “transesophageal echocardiography”, “intracardiac echocardiography”, “antithrombotic therapy”, “pulsed field ablation”, and “artificial intelligence”. Clinical practice guidelines, randomized trials, prospective and retrospective registries, comparative observational studies, systematic reviews, meta-analyses, imaging studies, and reports of emerging technologies were considered when they contributed directly to the thematic scope of the review. Preprints, conference abstracts, and early feasibility studies were included selectively when they addressed rapidly evolving areas not adequately represented by mature comparative evidence and were explicitly interpreted as preliminary evidence.

Evidence was interpreted according to methodological design and clinical relevance. Randomized controlled trials were assigned the greatest weight for comparative efficacy and safety because randomization provides the strongest protection against measured and unmeasured confounding. Systematic reviews and meta-analyses were used to assess consistency across studies but were interpreted according to the design, quality, and heterogeneity of their constituent studies and were not treated as evidence independent of, or inherently superior to, the underlying trials. Prospective registries were used primarily to characterize procedural performance, uncommon complications, and outcomes in broader clinical practice. Retrospective cohorts, propensity-matched analyses, and administrative-database studies were regarded as hypothesis-generating because residual confounding and treatment-selection bias cannot be excluded. Preprints, conference abstracts, early feasibility studies, and experimental investigations were considered preliminary and were not used alone to support definitive clinical recommendations.

Evidence selection was purposive and was based on clinical relevance, methodological importance, recency, and contribution to the review’s thematic framework. The search was not intended to identify every potentially eligible publication. No protocol was registered, and no duplicate independent screening, formal standardized study-level data-extraction protocol, PRISMA flow diagram, formal risk-of-bias assessment, certainty-of-evidence grading, or quantitative synthesis was undertaken. Accordingly, this article should be interpreted as a narrative state-of-the-art synthesis rather than a systematic or scoping review. Table 1 summarizes selected randomized trials informing the comparative evidence for LAAC, while registry, observational, meta-analytic, and emerging evidence is discussed according to its methodological context throughout the article.

## 3. LAAC as an Alternative to Oral Anticoagulation

Oral anticoagulation, particularly DOAC therapy, remains the standard first-line strategy for stroke prevention in nonvalvular AF. However, its long-term use may be limited by major bleeding risk, prior hemorrhage, frailty, renal dysfunction, intolerance, cost, and adherence challenges; patient preference may also influence shared decision-making but should not be equated with a contraindication to anticoagulation. LAAC has therefore emerged as a non-pharmacological stroke-prevention strategy that targets the predominant site of thrombus formation in nonvalvular AF. Current evidence suggests that LAAC is not a universal replacement for anticoagulation, but rather a patient-specific alternative in selected individuals.

### 3.1. Guideline Recommendations

The 2025 Society for Cardiovascular Angiography and Interventions/Heart Rhythm Society (SCAI/HRS) clinical practice guideline suggests LAAC over no therapy in patients with nonvalvular AF and contraindications to OAC. It also recognizes that either OAC or LAAC may be appropriate in selected patients pursuing stroke prevention, particularly when elevated bleeding risk, prior bleeding complications, or a strong preference to avoid long-term anticoagulation influences shared decision-making [1].

The 2024 European Society of Cardiology (ESC) guideline likewise states that percutaneous LAAC may be considered in patients with AF and contraindications to long-term anticoagulant treatment, while emphasizing that DOACs remain the standard treatment for anticoagulation-eligible patients and that the role of LAAC relative to contemporary DOAC therapy remains incompletely defined [22]. Overall, both North American and European guidance support individualized use of LAAC rather than routine replacement of anticoagulation, with decisions based on thromboembolic risk, bleeding risk, suitability for OAC, procedural risk, and patient preference [1,22].

### 3.2. Randomized Evidence: Context, Trial Design, and Generalizability

Early randomized evidence came from PROTECT-AF and PREVAIL, which compared the first-generation Watchman device with warfarin in patients with nonvalvular atrial fibrillation who were eligible for anticoagulation [8,9]. PROTECT-AF established noninferiority for its composite efficacy endpoint. PREVAIL did not meet the prespecified noninferiority criterion for its first coprimary composite endpoint, but it did establish noninferiority for post-procedural ischemic stroke or systemic embolism and showed improved early procedural safety compared with PROTECT-AF. These trials established the clinical foundation for percutaneous LAAC, but their use of warfarin and an earlier device generation limits direct applicability to contemporary DOAC-based practice.

OPTION evaluated LAAC specifically after atrial fibrillation ablation. The trial randomized 1600 patients with elevated thromboembolic risk to Watchman FLX LAAC or continued oral anticoagulation [13]. At 36 months, non-procedure-related major or clinically relevant nonmajor bleeding occurred in 8.5% of the LAAC group and 18.1% of the anticoagulation group, while the composite of all-cause death, stroke, or systemic embolism occurred in 5.3% and 5.8%, respectively. Device- or procedure-related complications occurred in 23 patients. Because procedure-related bleeding was excluded from the primary safety endpoint and the population was selected, anticoagulation-eligible, and undergoing or recently undergoing ablation, the findings should not be extrapolated automatically to patients outside this setting.

CHAMPION-AF randomized 3000 anticoagulation-eligible patients with nonvalvular atrial fibrillation to Watchman FLX LAAC or non-vitamin K antagonist oral anticoagulant therapy [18]. At three years, the composite of cardiovascular death, stroke, or systemic embolism occurred in 5.7% and 4.8% of patients, respectively, establishing noninferiority, while non-procedure-related bleeding occurred in 10.9% and 19.0%. Interpretation should account for the composite efficacy endpoint and the exclusion of procedure-related bleeding from the primary safety endpoint; therefore, noninferiority should not be interpreted as equivalence for every individual outcome or patient subgroup.

CLOSURE-AF studied a substantially older and higher-risk population and produced a contrasting result [19]. The trial randomized 912 patients at high risks of both stroke and bleeding to catheter-based LAAC or physician-directed best medical care, including anticoagulation when appropriate. After a median follow-up of three years, the composite of stroke, systemic embolism, major bleeding, or cardiovascular or unexplained death occurred at rates of 16.8 and 13.3 events per 100 patient-years, respectively, and noninferiority was not established. This finding limits extrapolation of the favorable OPTION and CHAMPION-AF results to older, frailer, and more multimorbid patients. Statistical design also differed across contemporary trials: CHAMPION-AF used an absolute noninferiority margin, whereas CLOSURE-AF used a hazard-ratio-based noninferiority framework; together with differences in endpoint composition and treatment of procedure-related events, these design choices affect the interpretation and cross-trial comparability of their findings [18,19].

These trials are not interchangeable. They differed in population, comparator therapy, device generation, endpoint composition, handling of procedure-related events, and noninferiority framework. Secondary evidence syntheses are summarized in Table 2. Across meta-analyses, LAAC has generally shown thromboembolic efficacy broadly comparable with oral anticoagulation, whereas estimates for bleeding and mortality vary according to the comparator, inclusion of procedure-related events, and the design of the constituent studies [23,24,25,26]. Mortality signals should be interpreted cautiously because some analyses include comparisons with warfarin or observational studies and therefore do not establish a mortality advantage over contemporary DOAC therapy [23,24,25]. Device-focused network analyses address a different question from LAAC-versus-anticoagulation comparisons [27], while network and reconstructed individual-patient-data analyses provide additional indirect comparative evidence [28], and the DOAC-focused preprint remains preliminary [29]. Overall, these secondary syntheses should be interpreted in the context of the underlying randomized evidence rather than as independent evidence of superiority.

### 3.3. Evidence Across Distinct Clinical Populations

Observational evidence broadens the clinical context of LAAC but should be interpreted below randomized evidence. In a six-center Italian cohort of 428 patients, ischemic stroke and major bleeding each occurred in 0.7% during a mean follow-up of 523 ± 58 days, supporting the feasibility of LAAC in routine practice while remaining observational evidence [30]. The OCEAN-LAAC registry further demonstrated that procedural results may influence subsequent outcomes, as peri-device leak was associated with a higher risk of transient ischemic attack, ischemic stroke, or systemic embolism, including with leaks of 3 mm or smaller [31]. These studies inform real-world feasibility and procedural quality but should not be interpreted as randomized evidence of comparative effectiveness.

#### 3.3.1. Anticoagulation-Ineligible Patients

Patients who are unable to receive long-term anticoagulation represent an important but incompletely studied LAAC population. COMPARE-LAAO randomized high-stroke-risk patients considered ineligible for anticoagulation to LAAC or standard care, but premature termination resulted in insufficient statistical power for definitive comparative conclusions [17]. This population should therefore be distinguished from anticoagulation-eligible participants enrolled in contemporary randomized trials. Patients who refuse OAC constitute a further distinct group rather than being pharmacologically ineligible. In the LAAC-REFUSAL study, OAC refusal accounted for only 4.5% of LAAC procedures; LAAC was technically feasible in carefully selected patients, although the absence of randomized comparison with DOAC therapy and the requirement for post-procedural antithrombotic treatment remain important considerations [32].

#### 3.3.2. Post-Ablation Patients

Patients undergoing or recently having undergone AF ablation constitute a specific population represented by OPTION and should not be generalized automatically to the broader AF population [13]. In secondary analyses, LAAC performed either concomitantly with or sequentially after ablation showed comparable efficacy to OAC while reducing clinically important bleeding [14]. Additional analyses found comparable AF recurrence after LAAC and showed that thromboembolic risk persisted even in patients without documented AF recurrence, supporting continued stroke-prevention strategies irrespective of rhythm status [15]. A sex-based analysis similarly found reduced non-procedural bleeding with LAAC without an apparent increase in thromboembolic risk in either sex [16].

Three distinct clinical questions should be separated in the post-ablation setting. First, discontinuation of long-term OAC after apparently successful AF ablation concerns thromboembolic-risk management after rhythm control and is not equivalent to LAAC. A 2026 systematic review and meta-analysis of 28 studies involving 267,443 patients found that OAC discontinuation after successful AF ablation was associated with less major bleeding without a statistically significant increase in thromboembolic events, although randomized confirmation and individualized patient selection remain necessary [33]. Second, OPTION addresses the separate question of whether LAAC can replace continued long-term OAC in selected anticoagulation-eligible patients after AF ablation [13]. Third, patients undergoing LAAC require a separate short-term post-implant antithrombotic strategy during device healing. Rhythm success, LAAC as an alternative to long-term OAC, and post-LAAC antithrombotic therapy should therefore not be considered interchangeable clinical decisions.

#### 3.3.3. Anticoagulation-Eligible Patients

Anticoagulation-eligible patients represent a clinically different population because LAAC is considered as an alternative to, rather than a substitute necessitated by the inability to receive OAC. CHAMPION-AF directly evaluated Watchman FLX against DOAC therapy in anticoagulation-eligible patients and established noninferiority for its composite efficacy endpoint while reducing non-procedural bleeding [18]. These findings should nevertheless be interpreted within the trial’s population, endpoint composition, and handling of procedure-related events, as discussed in Section 3.2, and should not be extrapolated to patients with contraindications to anticoagulation or substantially greater frailty and bleeding risk.

#### 3.3.4. Patients with Prior Major Bleeding or High Bleeding Risk

Patients with previous major bleeding or high predicted bleeding risk are frequently referred for LAAC but are less well represented in contemporary randomized comparisons. In a cardinality-matched high-bleeding-risk cohort, LAAC was associated with similar long-term rates of stroke and cardiovascular death but lower major bleeding than DOAC therapy [34]. In the retrospective multicenter PERSEO registry, patients undergoing LAAC after a severe bleeding event had better 24-month event-free and overall survival than those receiving medical management [35]. These observational associations are clinically relevant but remain susceptible to treatment-selection bias and residual confounding; in particular, they should not be interpreted as establishing a causal mortality benefit of LAAC.

#### 3.3.5. Prior Ischemic Stroke and Stroke Despite Adequate Anticoagulation

Patients with a history of ischemic stroke should be distinguished from the narrower population experiencing stroke despite verified adequate anticoagulation. In a propensity-matched TriNetX analysis of 3368 patients per group with prior ischemic stroke, LAAC was associated with lower risks of recurrent ischemic stroke and systemic embolism than DOAC therapy, together with an observed association with lower all-cause mortality [36]. Because the study was observational, residual confounding limits causal interpretation, particularly for mortality. Importantly, a history of ischemic stroke does not establish that the index stroke occurred despite adequate therapeutic anticoagulation. Evidence specifically supporting LAAC in patients with ischemic stroke despite confirmed appropriate OAC remains limited, and this population should therefore be considered a distinct evidence gap rather than inferred from studies enrolling patients with prior stroke.

### 3.4. Ongoing Investigation and Evidence Gaps

Important evidence gaps remain in anticoagulation-ineligible patients and post-procedural antithrombotic management. COMPARE-LAAO was terminated early and was underpowered, limiting firm conclusions in high-stroke-risk patients unable to receive oral anticoagulation [17]. ATLAAC is testing whether discontinuing oral anticoagulation after successful surgical LAAC is noninferior to continued anticoagulation for the composite of ischemic stroke, systemic arterial embolism, or major bleeding [20]. Although CHAMPION-AF has reported its 3-year primary results, its prespecified follow-up continues through 5 years, including a 5-year noninferiority endpoint of ischemic stroke or systemic embolism; these longer-term data remain important for defining durability and patient selection [37].

## 4. Contemporary LAAC Devices and Closure Strategies

LAAC has evolved from a niche option for patients unable to tolerate OAC into an important component of stroke prevention in nonvalvular AF. Contemporary strategies include endocardial occlusion, epicardial exclusion, surgical appendage closure, hybrid approaches, imaging-guided workflows, and selected combined ablation-LAAC procedures. Innovation has focused on procedural safety, PDL reduction, device-related thrombus (DRT) prevention, and suitability for complex LAA morphologies [2,3].

Current practice is dominated by the Watchman (Boston Scientific Corporation, Marlborough, MA, USA) and Amplatzer Amulet platforms (Abbott, Abbott Park, IL, USA). These platforms differ in anchoring, sealing, and post-procedural antithrombotic strategy [2,3,11]. LARIAT (SentreHEART, Inc., Redwood City, CA, USA) and AtriClip (AtriCure, Inc., Mason, OH, USA) provide alternatives in selected patients with challenging anatomy, contraindications to intracardiac foreign material, or need for surgical or hybrid AF management [4,5].

### 4.1. Endocardial Closure Strategies

#### 4.1.1. The Watchman Platform

Watchman 2.5 established the early randomized evidence base for percutaneous LAAC compared with warfarin in PROTECT-AF and PREVAIL [8,9]. Because these trials used warfarin as the comparator, their findings should not be interpreted as establishing a mortality advantage over contemporary DOAC therapy.

Watchman FLX and FLX Pro represent important engineering refinements. The FLX platform incorporates a rounded distal frame, increased recapturability, shorter device length, and a broader size matrix to improve conformability across diverse LAA anatomies [2,3]. These modifications were developed to reduce perforation, embolization, and residual PDL. Mechanistically, Watchman functions as a single-seal endocardial occluder positioned within the appendage ostium, with endothelialization over the polyethylene terephthalate membrane isolating the LAA from systemic circulation. Despite high efficacy, incomplete sealing remains relevant in trabeculated or multilobed appendages [2,11].

Contemporary registries report excellent implantation success and low DRT rates with newer-generation Watchman devices, supporting widespread clinical adoption [2,3]. However, short-term post-procedural anticoagulation may remain a limitation in patients with extreme bleeding risk. Recent strategies increasingly combine Watchman implantation with catheter ablation as a “one-stop” AF management approach in selected patients. However, the clinical value of combined procedures should be assessed against patient-specific rhythm-control indications and procedural risk [2].

#### 4.1.2. The Amplatzer Amulet Platform

The Amplatzer Amulet device introduced a dual-seal concept consisting of a distal lobe for anchoring and a proximal disc that seals the ostium externally [11]. This architecture improves ostial coverage and may reduce residual peri-device flow, particularly in complex appendages. The pivotal Amulet IDE trial demonstrated non-inferiority to Watchman for stroke prevention and major safety endpoints while achieving superior complete LAA occlusion rates [11].

This enhanced sealing may be accompanied by greater procedural complexity and higher rates of pericardial effusion or procedure-related complications, although operator experience may mitigate these risks [5,11]. Because Amulet provides immediate ostial coverage, it is often paired with dual antiplatelet therapy rather than transient OAC, making it attractive in patients with absolute anticoagulation intolerance [3,11]. Anatomically, it may suit shallow appendages, wide ostia, or multilobar morphologies [5,11].

### 4.2. Epicardial and Hybrid Closure Approaches

#### 4.2.1. LARIAT Suture Delivery System

The LARIAT device uses epicardial ligation through combined transseptal and pericardial access, excluding the LAA without an intracardiac implant [4,5]. This may reduce DRT risk and prolonged antithrombotic exposure, but implantation is technically demanding and associated with pericarditis, pericardial effusion, and access-related injury [4,5]. Selected indications include extremely large appendages, unsuitable landing zones, or failed endocardial closure.

#### 4.2.2. AtriClip and Surgical Exclusion

AtriClip is used during concomitant cardiac surgery or minimally invasive thoracoscopic AF procedures to externally exclude the appendage [5]. LAAOS III demonstrated stroke reduction when surgical LAA closure was added during cardiac surgery in patients with AF [10]. This supports surgical LAA exclusion as an adjunctive strategy during indicated cardiac surgery, not as direct evidence that anticoagulation can be discontinued. Thoracoscopic stand-alone AtriClip and hybrid epicardial ablation-clipping approaches may have a role in selected patients.

### 4.3. Emerging and Niche LAAC Technologies

Although Watchman and Amulet dominate current practice, several next-generation devices are under investigation. These technologies aim to improve anatomical adaptability, reduce thrombogenicity, simplify implantation, and expand the range of treatable LAA morphologies. Their current roles remain less established than those of Watchman and Amulet, and their long-term clinical value requires further comparative data.

The LAmbre device (Lifetech Scientific (Shenzhen) Co., Ltd., Shenzhen, China) uses a flexible umbrella-and-cover configuration. Its self-expanding nitinol umbrella anchors within the appendage, while a larger cover seals the ostium externally, providing adaptability to shallow, irregular, or multilobed appendages [5]. Multiple size configurations may allow treatment of appendages with marked discrepancies between ostial and landing-zone dimensions [5].

The WaveCrest occluder (Coherex Medical, Inc., Salt Lake City, UT, USA) features a self-expanding nitinol frame covered by an expanded polytetrafluoroethylene membrane. Its low-profile design is intended to minimize PDL and thrombogenicity while permitting repositioning, but large randomized data remain limited [5].

The Conformal Left Atrial Appendage Seal (CLAAS; Conformal Medical, Inc., Nashua, NH, USA) system represents a novel approach. Instead of relying primarily on radial force, CLAAS uses a compliant foam-based structure supported by a nitinol endoskeleton that passively conforms to LAA anatomy. This may reduce chronic wall stress, erosion, perforation, and pericardial effusion [38]. Its simplified two-size strategy may reduce procedural complexity, and early feasibility studies have shown successful implantation, favorable sealing, and low major complication rates [38]. The principal design characteristics, advantages, limitations, anatomical considerations, and evidence maturity of contemporary LAAC devices and closure strategies are summarized in Table 3.

## 5. Watchman Versus Amulet: Device Selection Beyond Superiority

### 5.1. Comparative Clinical Performance

The evolution of LAAC has shifted the discussion from identifying a universally superior device toward determining which platform is best suited to an individual patient. Watchman and Amplatzer Amulet have demonstrated broadly comparable efficacy for preventing stroke and systemic embolism in nonvalvular AF. Contemporary device selection is therefore increasingly guided by anatomy, procedural characteristics, post-procedural antithrombotic requirements, and operator experience rather than assumptions of overall superiority.

The randomized Amulet IDE trial showed that Amulet achieved non-inferior safety and effectiveness outcomes compared with Watchman while providing a higher rate of complete LAA occlusion at 45 days [11]. SWISS-APERO similarly reported comparable clinical outcomes, although Amulet was associated with lower PDL rates and Watchman with a more favorable procedural safety profile [12]. These findings suggest that differences between platforms relate more to anatomical and procedural performance than to major differences in clinical efficacy.

### 5.2. Device Design and Anatomical Considerations

**Figure 1:**
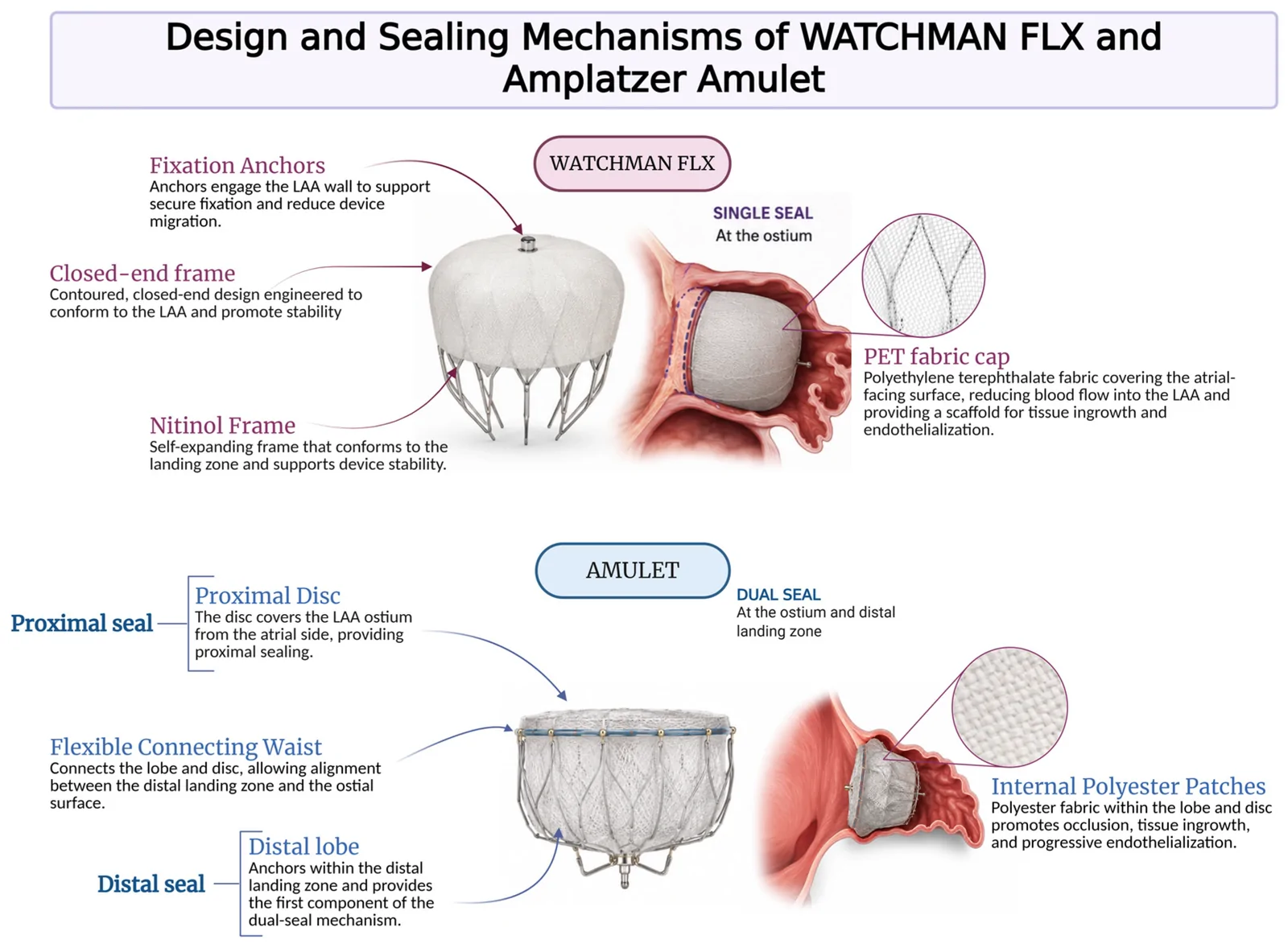
WATCHMAN FLX uses a single-seal, plug-based design positioned within the LAA landing zone, with fixation anchors, a self-expanding nitinol frame, and an atrial-facing PET fabric surface. Amplatzer Amulet uses a dual-seal design comprising a distal anchoring lobe and a proximal disc that covers the LAA ostium. These structural differences influence anatomical suitability, implantation technique, ostial coverage, and device selection. Device choice should be individualized according to ostial dimensions, landing-zone depth, appendage morphology, procedural considerations, and operator experience. Abbreviations: LAA, left atrial appendage; PET, polyethylene terephthalate.

Device design explains many observed procedural differences. Amulet uses a dual-seal configuration that may improve ostial coverage in large ostia, shallow landing zones, or complex lobar anatomies [11,12]. Watchman relies on a single-seal mechanism and is generally associated with simpler implantation, greater procedural familiarity, and lower rates of complications, such as pericardial effusion and device embolization [11,43]. Although Amulet may reduce PDL, the long-term clinical significance and optimal management of small residual leaks remain incompletely defined [5,12]. The principal structural and sealing differences between WATCHMAN FLX and Amplatzer Amulet are illustrated in Figure 1.

### 5.3. Long-Term Safety and Device-Related Complications

Comparative analyses support broadly similar long-term outcomes. A 2025 network meta-analysis of six randomized trials found no significant differences between Amulet and Watchman in stroke, mortality, thromboembolic events, or device embolization, although Amulet was associated with more pericardial effusion [27]. A 2024 meta-analysis similarly found no significant differences in stroke, cardiovascular mortality, thromboembolism, or major bleeding [44].

DRT remains clinically important. Dukkipati et al. reported a DRT incidence of approximately 3.7% after Watchman implantation, with higher thromboembolic event rates during follow-up [45]. This emphasizes careful technique, imaging surveillance, and individualized antithrombotic therapy. DOAC-based strategies, particularly low-dose regimens, may reduce thromboembolic and bleeding complications, although optimal selection remains unresolved [46].

### 5.4. Toward Personalized Device Selection

Current evidence does not establish universal clinical superiority of either Watchman or Amulet. Device choice should therefore remain individualized: Watchman may be favored when procedural simplicity, recapturability, and operator familiarity are priorities, whereas Amulet may be favored when broader ostial coverage is desirable, such as in shallow appendages, wide ostia, or selected complex morphologies. Differences in sealing performance or procedural complications should not be assumed to translate into differences in stroke prevention, mortality, or overall net clinical benefit because comparative evidence for these outcomes remains limited. Successful LAAC therefore depends on matching device characteristics to anatomy, procedural risk, bleeding profile, anticipated antithrombotic therapy, and operator experience [4,5,11,12,27,44].

## 6. Device Evolution and Complication Reduction: From the Plug Era to the Seal Era

### 6.1. From Mechanical Occlusion to Optimized Sealing

LAAC has evolved from simple mechanical exclusion toward anatomically precise and biologically optimized sealing. Contemporary devices are designed to achieve stable anchoring, complete closure, minimal PDL, low thrombogenicity, atraumatic deployment, and durable endothelialization. Meta-analytic evidence supports LAAC as an alternative to OAC in selected AF patients, providing comparable stroke prevention while potentially reducing hemorrhagic stroke and bleeding [23,24].

### 6.2. Watchman 2.5 and Percutaneous LAAC

Watchman 2.5 established the feasibility and clinical credibility of percutaneous LAAC. In PROTECT-AF and PREVAIL, Watchman was compared with warfarin and provided the early randomized evidence base for percutaneous LAAC [8,9]. Earlier-generation limitations included more challenging implantation in complex LAA anatomy, peri-device leaks, device recapture, and device-related thrombus, motivating subsequent device redesign [40].

### 6.3. Watchman FLX and FLX Pro

Watchman FLX addressed earlier limitations through 18 fixation anchors, a closed distal end, reduced atrial metal exposure, and a wider compression range, improving recapturability, stability, and anatomical adaptability [2,3,40]. In a meta-analysis of five retrospective observational studies including 54,727 patients, FLX was associated with higher procedural success and lower observed rates of mortality, major bleeding, device embolization, and pericardial effusion than Watchman 2.5, while stroke and DRT rates were similar [40]. Because these data were non-randomized, the observed mortality difference should not be interpreted as a causal mortality benefit.

Watchman FLX Pro further emphasizes biological healing through enhanced visibility, accommodation of larger anatomies, and a polymer-coated surface designed to promote endothelial healing and reduce thrombogenicity [2,3]. Clinical outcome evidence specific to FLX Pro remains less mature than the evidence base for the established FLX platform.

### 6.4. Amulet and Dual-Seal Closure

Amulet adopts a different approach by separating anchoring from ostial sealing [11]. An updated 2025 meta-analysis comparing Watchman FLX with Amulet found similar rates of PDL, DRT, and pericardial effusion, while stroke or transient ischemic attack was lower with Amulet; because the synthesis included one randomized trial and four cohort studies, these findings should be interpreted cautiously [41]. A network meta-analysis of randomized trials found similar stroke, mortality, thromboembolism, and device-embolization outcomes between Amulet and Watchman, but a higher pericardial-effusion risk with Amulet [27]. These findings reinforce individualized device selection based on anatomy and procedural priorities rather than device superiority alone.

### 6.5. PDL, DRT, and Imaging-Guided Reduction

Peri-device leak (PDL) and device-related thrombus (DRT) are clinically relevant findings after LAAC, but their interpretation requires distinction between observational association and proven treatment benefit. Residual LAA patency and PDL have been associated with increased thromboembolic risk, although the magnitude of risk varies according to leak size, imaging modality, device type, patient characteristics, and antithrombotic treatment [47,48]. Importantly, these associations do not establish that eliminating or treating every small residual leak necessarily reduces subsequent stroke or systemic embolism.

Similarly, DRT is associated with increased thromboembolic risk and appears to be influenced by device positioning, residual flow, endothelial healing, and patient-level thrombotic factors [45,49,50,51]. Antithrombotic strategies may modify DRT and bleeding risk, but available comparative evidence does not establish a single universally superior preventive regimen. Pre-procedural CT and procedural imaging may improve anatomical assessment, device sizing, and implantation efficiency [52]; however, improvements in procedural or imaging endpoints should not automatically be interpreted as proven reductions in major clinical events. These findings support anatomy-guided implantation and structured surveillance while highlighting the need for prospective evidence linking procedural optimization to clinically meaningful outcomes.

Terminology should also reflect the imaging modality and mechanism of residual flow. On TEE, peri-device leak generally refers to residual flow around the device margin, whereas CT may demonstrate the broader finding of residual LAA patency caused by peri-device communication, incomplete coverage or an uncovered lobe, device malapposition, or trans-fabric permeability. Peri-device leak and residual LAA patency should therefore not be used interchangeably. Similarly, CT-detected hypoattenuated thickening may represent expected device healing when low grade, whereas protruding or pedunculated high-grade hypoattenuated thickening is more consistent with definite DRT. Reported 3 mm and 5 mm leak thresholds should be interpreted according to imaging modality, device type, assessment timing, and study definition. Follow-up imaging is commonly performed approximately 45–90 days after LAAC, with additional evaluation guided by clinical or imaging findings. Although larger residual leaks and definite DRT are associated with thromboembolic risk, evidence remains insufficient to establish that antithrombotic escalation or repeat closure of every small residual abnormality improves clinical outcomes [47,48,51,53].

## 7. Imaging-Guided Strategies in LAAC

### 7.1. Pre-Procedural Planning: The Expanding Role of CT

Pre-procedural imaging supports thrombus exclusion, anatomical characterization, device sizing, and planning. Although TEE has traditionally served as the reference standard, cardiac CT provides three-dimensional assessment of LAA morphology, ostial diameter, landing-zone dimensions, and depth [6,52]. Virtual implantation can reduce device changes and procedure time, and CT can exclude LAA thrombus when contrast filling is complete, although equivocal findings require TEE confirmation [6,52]. The 2025 SCAI/HRS guideline recommends baseline imaging with either TEE or CT [1].

Beyond LAA anatomy, cardiac magnetic resonance (CMR), particularly late-gadolinium-enhancement imaging, can characterize atrial fibrosis and atrial cardiomyopathy and may provide complementary information regarding AF substrate, particularly when LAAC is considered alongside rhythm-control strategies [54]. However, CMR-derived atrial substrate measures have not been validated as routine criteria for LAAC candidacy, device selection, or post-procedural surveillance; their current role in the LAAC pathway therefore remains complementary rather than established.

### 7.2. Intraprocedural Guidance: TEE Versus Intracardiac Echocardiography

Intraprocedural imaging is essential for safe device deployment. TEE provides high-resolution real-time imaging but often requires general anesthesia and carries a risk of esophageal injury. Intracardiac echocardiography (ICE) has emerged as an alternative that allows LAAC under local anesthesia as part of a more minimalist approach. In a 2025 meta-analysis of 19 studies including 44,706 patients, ICE was associated with slightly higher procedural success but higher pericardial effusion risk [55]. Another 2025 meta-analysis of 18 studies including 124,230 patients found that ICE improved technical success and reduced device use, but increased pericardial effusion or tamponade, iatrogenic atrial septal defects, and vascular complications [56].

An updated 2026 meta-analysis adjusted for confounders showed that ICE shortened procedure time while maintaining comparable procedural success, but increased intervention-requiring pericardial effusion and residual iatrogenic atrial septal defect [57]. This risk may reflect greater catheter manipulation and the need for additional transseptal access. Importantly, complication rates appear to decrease with operator experience [57]. ICE is therefore an effective alternative to TEE, especially when general anesthesia is undesirable, but requires awareness of the learning curve and mechanical risks.

### 7.3. Post-Procedural Surveillance: CT Versus TEE

Post-procedural imaging is recommended 45–90 days after LAAC to assess PDL, DRT, and device position [1]. TEE has traditionally been used for surveillance, but CT offers superior spatial resolution. A 2025 meta-analysis of 17 studies including 2036 patients reported that CT detected residual LAA patency in 54.6% and PDL in 51.5% [47]. CT-detected LAA patency was associated with almost two-fold increased thromboembolic risk, while Bayesian analysis showed a 98.5% probability that residual patency increases risk [47]. A 2025 conference abstract reported higher PDL detection with CT than TEE, although CT involves radiation and contrast exposure [58]. These findings support CT as a valuable surveillance tool when precise leak characterization is needed. Management decisions should integrate CT findings with TEE findings, leak size, device type, bleeding risk, thrombotic risk, and the feasibility of continued antithrombotic therapy.

### 7.4. Emerging Frontiers: Artificial Intelligence, Photon-Counting CT, and Fusion Imaging

Artificial intelligence (AI)-assisted three-dimensional heart models derived from intraprocedural TEE can automatically segment the LAA and provide real-time measurements. In a prospective study of 66 patients, automated landing-zone measurements showed good agreement with TEE and angiography, while predicted implantation angulations were consistent with CT-based planning [59]. Although the prototype underestimated some measurements compared with multidetector CT, it was safe, feasible, and reduced the need for multiple device deployments [59].

Photon-counting CT (PCCT) provides ultra-high spatial resolution and reduces metallic artifacts. A 2025 case series showed that PCCT can identify mechanisms of PDL, including fabric deficiency, off-axis positioning, and low-volume residual contrast ingress, potentially guiding decisions such as device repositioning or continued anticoagulation [60]. PCCT remains an early-stage adjunct and should be validated according to procedural efficiency, safety, and clinical outcomes rather than imaging performance alone.

Overall, the evidence supporting multimodality imaging is strongest for anatomical characterization, procedural guidance, detection of residual abnormalities, and workflow optimization. Much of the comparative literature evaluates technical success, procedural efficiency, or imaging-detected endpoints rather than stroke, mortality, or other definitive clinical outcomes. Consequently, greater sensitivity for detecting PDL or DRT, more accurate device sizing, or shorter procedure times should not in themselves be interpreted as proof of improved long-term clinical outcomes. Imaging strategies should therefore be selected according to their established procedural role, patient characteristics, local expertise, and the clinical relevance of the information obtained. The roles, strengths, limitations, and potential management implications of multimodality imaging across the LAAC pathway are summarized in Table 4.

## 8. Optimizing Procedural Outcomes

### 8.1. Standardizing Evaluation Criteria and Imaging Modalities

Optimizing LAAC outcomes requires standardized post-closure evaluation. Procedural success is commonly assessed by the absence of persistent PDL flow between the left atrium and LAA and by a residual stump or leak below 3–5 mm, depending on the criteria [53]. The timing and modality of imaging should be tailored to the evaluation stage. TEE remains central for perioperative and intraprocedural assessment because it guides deployment, sizing, repositioning, and immediate closure confirmation [53]. CT provides superior anatomical detail during follow-up, allowing assessment of residual stump morphology, device position, appendage patency, and endothelialization over the mid- to long-term period [6,53]. A standardized imaging vocabulary is needed so that PDL, residual patency, incomplete endothelialization, and DRT are defined consistently across studies and clinical practice.

### 8.2. Procedure Selection and In-Hospital Safety

Immediate procedural safety depends on matching patient risk with closure strategy. Endocardial occlusion devices account for most catheter-based LAAC procedures [61], but endocardial, epicardial, and surgical approaches involve different trade-offs. Comparative evidence suggests that catheter-based LAAC combined with ablation may achieve higher acute procedural success than thoracoscopic clipping in selected settings, but with different bleeding- and access-related trade-offs [62]. Approach selection should reflect ischemic risk, bleeding risk, anatomy, procedural suitability, and independent rhythm-control indications [61,62].

### 8.3. Concomitant Interventions

Combining catheter ablation and LAAC in a single procedure may provide procedural convenience by addressing rhythm control and stroke prevention during the same encounter [63,64]. Observational and early prospective studies suggest that concomitant procedures are feasible and can achieve high rates of pulmonary vein isolation and successful device implantation [63,64,65,66]. However, these findings largely reflect procedural and observational outcomes and do not establish that concomitant ablation–LAAC provides superior stroke prevention, survival, or long-term rhythm outcomes compared with appropriately selected standalone or staged procedures. Propensity-matched analyses have reported broadly comparable long-term outcomes between standalone LAAC and combined LAAC–ablation, but residual confounding cannot be excluded [64]. Accordingly, the decision to combine procedures should be driven primarily by an independent indication for rhythm control, patient anatomy, procedural suitability, and individualized risk rather than an expectation of additional thromboembolic or survival benefit.

### 8.4. Preventing Device-Related Thrombosis

DRT remains an important determinant of long-term procedural success [45,53]. Contemporary evidence suggests that DRT occurs in approximately 2–7% of patients and is associated with increased thromboembolic risk [45,53]. Risk factors include persistent or permanent AF, chronic kidney disease, diabetes mellitus, reduced left ventricular ejection fraction, and larger LAA dimensions [3,45,53]. Modifiable procedural determinants include device sizing, landing-zone positioning, avoidance of excessive implantation depth, and minimization of residual flow stagnation [3,53]. These findings emphasize meticulous planning, accurate deployment, individualized antithrombotic therapy, and structured imaging follow-up.

### 8.5. Implantation Technique and Device Compression

Implantation technique may influence residual sealing after LAAC. The IMPRESSION-LAAC study included 236 patients undergoing TEE-guided Watchman FLX implantation at two high-volume centers and compared device compression > 30% with compression ≤ 30% [67]. At 2-month TEE follow-up, residual PDL was less frequent with >30% compression (8.2% vs. 32.6%; *p* < 0.001), while procedural complications (1.6% vs. 1.7%) and the composite of cardiovascular death, ischemic stroke, or systemic embolism during follow-up (9.8% vs. 12.0%) did not differ significantly between groups [67]. Device compression was independently associated with residual PDL (adjusted OR, 0.935; 95% CI, 0.927–0.948; *p* = 0.003). These observational findings suggest that compression is an important technical determinant of sealing, but they do not establish that deliberate overcompression improves major clinical outcomes. Implantation should therefore balance adequate compression and sealing against device stability, anatomy, and procedural safety while awaiting prospective validation of clinically optimal compression targets [67].

### 8.6. CT-Based Planning and Advanced Sizing

Accurate screening and anatomical assessment identify contraindications such as LAA thrombus and reduce complications [6]. Although TEE remains important intraprocedurally, cardiac computed tomography angiography (CCTA) has become central to planning because of its spatial resolution and three-dimensional visualization [6]. It defines appendage morphology, ostial and landing-zone dimensions, depth, deployment angles, and relationships to adjacent structures, improving size selection, procedural success, recapture or exchange rates, and fluoroscopic projection planning [6,52].

## 9. Current Limitations and Unresolved Challenges

### 9.1. Post-Procedural Antithrombotic Regimens

A major unresolved challenge is the absence of a universally established post-procedural antithrombotic regimen. Therapy is intended to reduce thrombotic risk during device endothelialization, but LAAC populations differ substantially in bleeding risk, anticoagulation tolerance, device type, and residual PDL or DRT [7]. Comparative studies and network meta-analyses suggest potential differences among warfarin, DOAC-based strategies, dual antiplatelet therapy, and reduced-dose regimens [3,7,46,49,68]. However, much of this evidence is indirect or non-randomized, and apparent reductions in bleeding or thrombotic events should not be interpreted as establishing one regimen as universally superior. Post-procedural therapy should therefore remain individualized until adequately powered randomized studies define the optimal strategy for specific patient and device populations.

### 9.2. Evidence Gaps in High-Risk Subgroups

Despite increasing adoption, the evidence base remains limited compared with modern DOAC therapy, especially in high-risk subgroups. Patients considered for LAAC often have prior major bleeding, renal dysfunction, advanced age, recurrent thromboembolism despite anticoagulation, or contraindications to oral anticoagulants. These populations are frequently underrepresented or heterogeneously defined in studies, leaving uncertainty regarding net clinical benefit over DOACs [69]. Persistent LAA thrombus despite adequate DOAC therapy also remains poorly defined, with no prospectively validated management strategy [70].

### 9.3. Heterogeneity and Comparative Uncertainty

Comparative evidence between LAAC and DOACs is limited by heterogeneity in study design, patient selection, device type, procedural experience, follow-up duration, and outcome definitions [23,24,25,29,69]. Much of the evidence comes from observational cohorts and registries, which are vulnerable to selection bias and confounding by indication. Patients referred for LAAC often differ clinically from those maintained on DOACs. Heterogeneity also extends to post-procedural anticoagulation; observational comparisons of DOACs and warfarin after LAAC illustrate variation in management strategies and outcome assessments [68].

Another unresolved issue is whether LAAC offers a clear advantage over contemporary DOAC therapy. Earlier evidence established LAAC primarily against warfarin, whereas DOACs now represent standard pharmacological therapy. Current randomized and pooled evidence suggests broadly comparable thromboembolic outcomes in selected populations, but superiority has not been consistently demonstrated across ischemic stroke, systemic embolism, cardiovascular death, all-cause mortality, or major bleeding [18,19,23,24,25,29]. Its role as a direct alternative in patients who can safely tolerate DOACs therefore remains uncertain and should be framed through shared decision-making rather than routine substitution [1,22].

### 9.4. Procedural Safety

**Figure 2:**
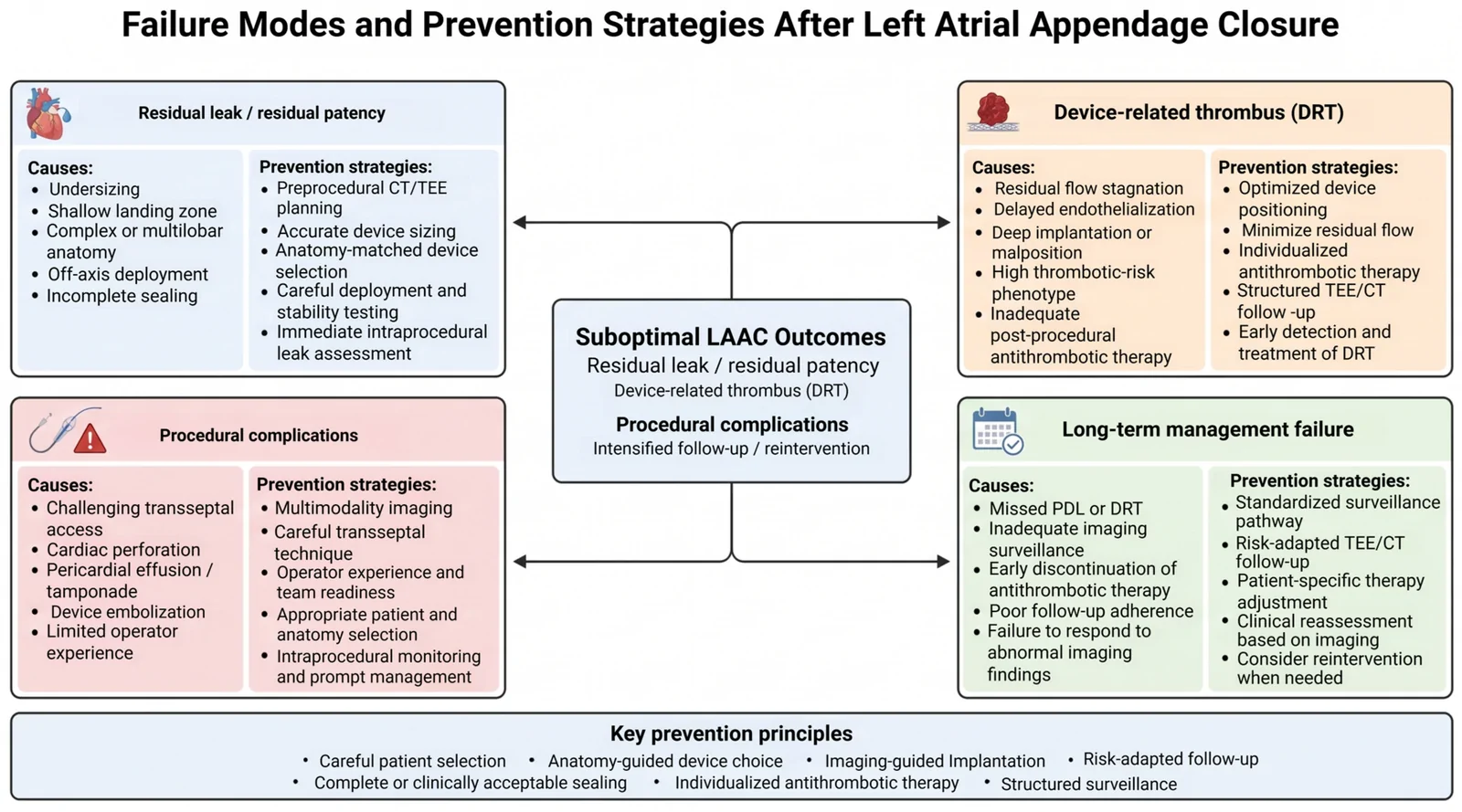
Suboptimal LAAC outcomes may result from residual leak or residual LAA patency, device-related thrombus, procedural complications, and inadequate long-term management. These risks may be reduced through careful patient selection, anatomy-guided device choice, multimodality imaging, optimized implantation and sealing, individualized antithrombotic therapy, and structured surveillance. Abbreviations: CT, computed tomography; DRT, device-related thrombus; LAAC, left atrial appendage closure; PDL, peri-device leak; TEE, transesophageal echocardiography.

Although procedural safety has improved, LAAC remains invasive. Complications include pericardial effusion or tamponade, intraoperative thrombosis, DRT, PDL, systemic embolism, bleeding, and device-related structural complications [71]. These events may require urgent intervention and affect both short-term safety and long-term efficacy. DRT is particularly important because it may require renewed anticoagulation, potentially undermining the rationale for LAAC in patients with bleeding contraindications [7,71]. Improved procedural standardization, structured imaging follow-up, and evidence-based post-procedural management remain essential. The principal failure modes across the LAAC pathway and their corresponding prevention strategies are summarized in Figure 2.

## 10. Emerging Techniques and Future Frontiers

Emerging approaches to LAAC differ substantially in evidentiary maturity and should not be interpreted as equivalent advances. For clinical interpretation, these technologies can be considered across three levels of evidence: (1) near-term clinical strategies, supported by comparative or prospective human data but still requiring further validation; (2) early clinical or feasibility-stage technologies, supported primarily by small prospective studies, case series, or first-in-human experience; and (3) experimental or preclinical technologies, for which clinical effectiveness remains unestablished. This hierarchy is used below to avoid attributing clinical benefit to technologies supported predominantly by feasibility or surrogate outcomes.

### 10.1. Magnetofluid Embolization and Bioresorbable Devices

Magnetofluid-based LAA occlusion represents an experimental preclinical strategy designed to achieve appendage exclusion without a permanent metallic implant. Preclinical studies have demonstrated feasibility and endothelialization, but no established human efficacy or safety evidence currently supports routine clinical use [72]. Bioresorbable occluders similarly aim to provide temporary structural support followed by scaffold degradation. Initial human experience suggests technical feasibility, but evidence remains limited and insufficient for comparison with the established Watchman or Amulet platforms. These approaches should therefore be considered investigational rather than near-term alternatives to contemporary LAAC.

### 10.2. Artificial Intelligence and Automated Planning

AI-assisted planning is being explored to improve procedural precision. Three-dimensional models derived from intraprocedural TEE can automatically segment the LAA and provide real-time measurements. In a prospective study of 66 patients, automated landing-zone measurements agreed with TEE and angiography, while predicted implantation angulations matched CT-based planning. This reduced multiple device deployments and demonstrated feasibility for real-time procedural support [59].

More broadly, AI applications in cardiac electrophysiology include signal interpretation, substrate characterization, risk prediction, procedural planning, and clinical decision support [73]. Within LAAC, future applications may include digital-twin simulation, CT-fluoroscopy fusion, deployment-angle prediction, and automated DRT or PDL risk assessment. However, algorithmic accuracy, successful segmentation, or improved procedural efficiency does not by itself establish clinical utility. External validation across populations and imaging platforms, prospective workflow evaluation, and demonstration of improved patient-relevant outcomes remain necessary. Accordingly, AI-assisted planning should currently be regarded as an early clinical decision-support technology; improved measurement or procedural efficiency has not yet been shown to improve major clinical outcomes.

### 10.3. Photon-Counting CT

PCCT offers higher spatial resolution, improved energy discrimination, and reduced metallic artifact compared with conventional CT. A 2025 case series showed that PCCT identified PDL mechanisms such as fabric deficiency, off-axis positioning, and low-volume residual flow [60]. It may support individualized anticoagulation, surveillance, or repeat intervention decisions, but remains an emerging adjunct rather than a standard surveillance modality. Accordingly, PCCT remains an early clinical imaging technology whose incremental diagnostic value has not yet been shown to translate into improved thromboembolic or survival outcomes.

### 10.4. Low-Dose DOAC Strategies

Post-procedural antithrombotic therapy remains unresolved. A 2025 network meta-analysis of 52 studies including 69,751 patients found that DOAC-based strategies had a favorable safety-efficacy profile compared with antiplatelet regimens [46]. Low-dose DOAC strategies were associated with lower major bleeding than standard-dose DOACs without apparent loss of thromboembolic or DRT protection [46]. These findings support individualized therapy but require prospective validation before any reduced-dose approach becomes standard. These findings remain hypothesis-generating and require prospective randomized validation before reduced-dose strategies can be considered established post-LAAC therapy.

### 10.5. Pulsed Field Ablation and LAAC

Pulsed field ablation (PFA), a non-thermal myocardial-selective energy source, has increased interest in same-session rhythm control and LAAC. A 2026 prospective single-center study of 209 patients found combined zero-fluoroscopy PFA and low-fluoroscopy LAAC feasible, with CT and AI-based planning used for device selection and adequate LAA sealing achieved in all patients [65]. Larger studies must define patient selection, safety, rhythm outcomes, and advantages over staged procedures [65]. PFA–LAAC should therefore be considered an early clinical combined-procedure strategy rather than an established approach with proven superiority over staged treatment.

### 10.6. Cryoballoon-Based LAA Isolation

Cryoballoon technology has been extended from pulmonary vein isolation to electrical LAA isolation. A 2026 prospective study of 23 patients with persistent AF found 91% acute isolation success, but 26% developed LAA thrombus on follow-up TEE despite therapeutic OAC [21]. Thrombus occurred only with durable LAA isolation, suggesting that electrical isolation without closure may create a pro-thrombotic environment through loss of LAA contractility. Timely mechanical closure and surveillance may therefore be required, but validation is needed. The small sample size and surrogate electrophysiological endpoints place this strategy at the early feasibility stage.

### 10.7. Epicardial Innovations and Hybrid Platforms

Epicardial-only systems continue to mature. The Sierra Aegis percutaneous subxiphoid ligation device achieved 100% procedural success in a first-in-human study of seven patients, with no leaks or DRT at one year [42]. Its zero-contrast, zero-heparin, and zero-implant profile may be useful in renal impairment, bleeding diathesis, or failed endocardial closure [42]. Totally thoracoscopic epicardial clip placement using AtriClip or Penditure (Medtronic, Minneapolis, MN, USA), combined with epicardial ablation as part of a total thoracoscopic maze (TT-MAZE) strategy, has also shown promising long-term outcomes, including complete closure and sustained sinus rhythm in selected patients [74]. These approaches remain early clinical or feasibility-stage strategies because current evidence is derived predominantly from small observational or first-in-human experiences rather than comparative randomized trials.

### 10.8. Endocardial Obliterative Purse-String Plication

Endocardial obliterative surgical closure represents another emerging refinement for patients already undergoing minimally invasive or robotic cardiac surgery. Unlike conventional orifice-only endocardial closure, which may leave a residual stump or small perfused cavity, inverted purse-string plication aims to obliterate the LAA lumen itself. In this technique, the distal half of the appendage is inverted into the left atrium, compacted with a purse-string suture along the pectinate muscle, restored toward its native orientation, and then closed with a two-layer orifice suture [75]. In an early retrospective series of 24 patients undergoing robotic mitral surgery or related minimally invasive procedures, postoperative electrocardiography (ECG)-gated CT demonstrated complete closure in all patients, with no residual contrast filling, pericardial effusion, periappendage hematoma, stroke, bleeding event, or left circumflex artery injury during early follow-up [75]. This approach may be attractive because it is inexpensive, avoids an intracardiac device, and directly targets the residual-cavity mechanism of incomplete closure. Accordingly, this technique should currently be considered an early surgical feasibility strategy; larger comparative studies and longer follow-up are required before conclusions regarding durability or thromboembolic benefit can be drawn.

## 11. Proposed Framework for Precision LAAC

### 11.1. Conceptual Basis

LAAC is evolving from a non-pharmacological alternative to OAC into a comprehensive patient-tailored stroke-prevention strategy. Successful outcomes depend not only on implantation but also on patient selection, anatomical assessment, imaging, device choice, implantation technique, antithrombotic therapy, and long-term surveillance [1,3]. Precision LAAC aims to standardize the care pathway while tailoring key decisions to individual patient characteristics [1,3].

### 11.2. Patient Selection and Shared Decision-Making

The first pillar is rigorous patient selection. LAAC should not be viewed as a universal OAC replacement, but as an option for patients with nonvalvular AF, high stroke risk, and unsuitability for long-term anticoagulation because of bleeding, frailty, recurrent falls, intolerance, or poor adherence [1,3]. The 2025 SCAI/HRS guideline supports LAAC in patients with OAC contraindications while recognizing that either OAC or LAAC may be appropriate in selected cases [1]. Shared decision-making is therefore essential [1,22].

### 11.3. Anatomy-Based Assessment and Device Selection

The second pillar is anatomy-based assessment. Appendage size, depth, angulation, lobar complexity, and ostial geometry influence device selection, procedural difficulty, sealing, and complications [3,5,6]. Pre-procedural imaging should define ostial diameter, landing-zone depth, appendage orientation, trabeculation, and adjacent structures to reduce residual leak or instability [5,6,52].

Device selection should then be anatomy-driven. Watchman FLX and FLX Pro offer recapturability, conformability, and procedural safety, whereas Amulet provides enhanced ostial coverage through dual-seal design [3,43]. Clinical outcomes are broadly comparable, but Amulet may reduce PDL in selected anatomies [43].

### 11.4. Integrated Imaging and Complete Sealing

Optimized imaging should be integrated throughout the LAAC pathway. Cardiac CT improves anatomical assessment and sizing [6,52], TEE or ICE guides transseptal puncture, positioning, stability testing, and leak assessment [1,55,56,57], and post-procedural imaging detects PDL, residual patency, DRT, migration, and incomplete endothelialization [47,48,53].

Complete sealing should remain a procedural goal. TEE-detected PDL is associated with increased thromboembolic risk, particularly with larger leaks [48], and CT-detected residual LAA patency has been linked to nearly double the odds of thromboembolism [47]. Management of small residual leaks should be individualized by leak size, imaging modality, thromboembolic risk, bleeding risk, and antithrombotic tolerance.

### 11.5. DRT Prevention and Long-Term Follow-Up

DRT remains a major complication influenced by device design, implantation depth, residual flow, endothelialization, and antithrombotic therapy [51,76]. Post-procedural therapy should therefore be individualized according to bleeding risk, thrombotic risk, device type, residual leak, and imaging findings [1,51,76]. Long-term surveillance remains necessary because late thrombus can occur even with minimal residual leaks [47,51,77]. Precision LAAC integrates patient selection, anatomy, imaging, device selection, individualized antithrombotic therapy, and structured follow-up into a unified stroke-prevention pathway.

## 12. Discussion

LAAC has evolved from a procedural alternative for patients unable to tolerate anticoagulation into an increasingly individualized strategy for stroke prevention in AF. Its comparative value remains population dependent and should be interpreted according to thromboembolic risk, bleeding risk, anticoagulation suitability, procedural risk, anatomy, and the strength of the supporting evidence rather than as a universal alternative to contemporary DOAC therapy.

Clinical success should be evaluated across the entire longitudinal pathway, including appropriate patient selection, anatomy-matched device and procedural strategy, effective sealing, safe endothelialization, post-procedural antithrombotic management, and structured imaging surveillance. Device, imaging, and procedural refinements may improve technical or surrogate outcomes, but these improvements should not automatically be interpreted as reductions in stroke, mortality, or other major clinical events without adequate comparative evidence.

Important uncertainties remain. Post-procedural antithrombotic therapy is not standardized, small PDLs remain incompletely understood, and comparative evidence against contemporary DOAC therapy continues to evolve. Many high-risk subgroups are also underrepresented in randomized trials. These gaps highlight the need for longer follow-up, standardized imaging definitions, and prospective studies in clearly defined patient populations.

### Review-Level Methodological Limitations

As a narrative review, this article has important methodological limitations. The literature search was targeted rather than exhaustive, and evidence selection was guided by author judgment rather than duplicate independent screening against predefined eligibility criteria. No review protocol was registered, and no formal risk-of-bias assessment or certainty-of-evidence grading was performed. Selective evidence inclusion, citation bias, and publication bias are therefore possible, particularly in rapidly evolving areas supported mainly by observational studies, early feasibility reports, conference data, or preprints. The conclusions should consequently be interpreted as a clinically oriented state-of-the-art synthesis rather than as a comprehensive systematic estimate of comparative effectiveness.

## 13. Future Directions

Future progress should focus on refining patient selection, standardizing post-procedural management, and improving procedural precision. Large randomized and pragmatic studies are needed to clarify which patients benefit most from LAAC compared with modern anticoagulation, particularly anticoagulation-eligible patients, those with prior ischemic stroke, chronic kidney disease, recurrent bleeding, frailty, or persistent LAA thrombus despite therapy.

Post-procedural antithrombotic therapy remains a major priority. Future studies should evaluate risk-adapted regimens based on bleeding risk, thrombotic risk, device type, residual leak, and imaging evidence of endothelialization. Low-dose DOAC strategies and imaging-guided discontinuation may be promising, but require prospective validation before routine use.

Technological development will also shape the next phase of LAAC, but advances should be prioritized by clinical readiness. Near-term priorities include CT-based planning, ICE-guided workflows, standardized surveillance, and risk-adapted antithrombotic regimens. Feasibility-stage tools include AI-assisted planning, automated segmentation, computational modeling, CT-fluoroscopy fusion, PCCT, conformable occlusion systems, and integrated PFA-LAAC pathways. Earlier-stage experimental concepts, including magnetofluid embolization and fully bioresorbable occluders, require validation before routine adoption. These innovations should be judged by clinically meaningful outcomes rather than feasibility alone.

Finally, integrated rhythm-control and stroke-prevention pathways may become increasingly relevant. Combined ablation and LAAC, particularly with PFA, may reduce procedural burden in selected patients. However, combined procedures should be guided by rhythm-control indications, anatomical suitability, bleeding risk, and individualized procedural risk rather than routine use in all patients.

## 14. Conclusions

LAAC has become an important component of contemporary stroke prevention in AF. Its greatest value lies not in replacing anticoagulation for all patients, but in offering an individualized alternative when long-term anticoagulation is unsuitable, poorly tolerated, or clinically uncertain. Advances in devices, imaging, procedural technique, and antithrombotic management are transforming LAAC into a more precise and patient-centered intervention.

The future of LAAC depends on matching each patient with the appropriate device, imaging strategy, procedural approach, antithrombotic regimen, and surveillance plan. As evidence matures, precision-guided LAAC is likely to play an increasing role in selected patients with AF.

## Figures and Tables

**Table 1:** Selected randomized trials informing the comparative evidence for left atrial appendage closure.

Study	Design	Population/Focus	Intervention/Comparator	Key Findings	Relevance to Precision-Guided LAAC
PROTECT-AF	Randomized controlled trial	Patients with nonvalvular AF eligible for warfarin	Watchman LAAC vs. warfarin	Demonstrated that percutaneous LAAC could provide stroke-prevention efficacy comparable to warfarin	Established the clinical foundation for percutaneous LAAC as an alternative stroke-prevention strategy [8]
PREVAIL	Randomized controlled trial	Patients with nonvalvular AF eligible for warfarin	Watchman LAAC vs. warfarin	Supported procedural safety and efficacy of Watchman LAAC, complementing PROTECT-AF	Reinforced early randomized evidence for LAAC and highlighted the importance of procedural experience [9]
LAAOS III	Randomized controlled trial	Patients with AF undergoing cardiac surgery	Surgical LAA occlusion plus usual care vs. usual care alone	Surgical LAA occlusion reduced stroke/systemic embolism when added during indicated cardiac surgery	Supports LAA exclusion as an adjunctive strategy during cardiac surgery, but not as direct evidence for stopping anticoagulation [10]
Amulet IDE	Randomized controlled trial	Patients with nonvalvular AF undergoing percutaneous LAAC	Amplatzer Amulet vs. Watchman	Amulet was non-inferior to Watchman for safety and effectiveness and achieved higher complete LAA occlusion at 45 days	Supports anatomy-based device selection and illustrates differences between dual-seal and single-seal platforms [11]
SWISS-APERO	Randomized clinical trial	Patients undergoing percutaneous LAAC	Amulet vs. Watchman	Reported comparable clinical outcomes, with lower peri-device leak rates for Amulet and procedural safety differences between devices	Strengthens the concept that device choice should be guided by anatomy, sealing goals, and procedural risk rather than universal superiority [12]
OPTION	Randomized controlled trial and secondary analysis	Anticoagulation-eligible patients with elevated thromboembolic risk undergoing or recently having undergone AF ablation	Watchman FLX LAAC vs. anticoagulation after ablation	LAAC reduced non-procedural bleeding and was noninferior to anticoagulation for death, stroke, or systemic embolism after AF ablation	Supports LAAC in selected post-ablation patients, but generalizability beyond this setting is limited [13,14,15,16]
COMPARE-LAAO	Randomized controlled trial, prematurely terminated	High-stroke-risk AF patients ineligible for anticoagulation	LAAC vs. standard care	Trial was underpowered because of early termination, limiting firm conclusions	Highlights the difficulty of generating randomized evidence in OAC-ineligible high-risk populations [17]
CHAMPION-AF	Multinational randomized controlled trial	Patients with nonvalvular AF considered suitable for long-term anticoagulation	Watchman FLX LAAC vs. DOAC therapy	LAAC was noninferior to DOACs for the composite efficacy endpoint and reduced non-procedural bleeding; ischemic stroke was numerically higher with LAAC	Supports LAAC in anticoagulation-eligible patients, but interpretation depends on endpoint composition, procedural-event handling, and trial design [18]
CLOSURE-AF	Multicenter randomized controlled trial	Predominantly older patients with AF at high risks of both stroke and bleeding	Catheter-based LAAC vs. physician-directed best medical therapy, including anticoagulation when appropriate	LAAC did not establish noninferiority to physician-directed medical therapy in patients at high risks of stroke and bleeding	Shows that favorable results from OPTION and CHAMPION-AF should not be extrapolated to older, higher-risk patients. [19]
ATLAAC	Randomized trial design/ongoing evidence gap	Patients after LAAC requiring post-procedural antithrombotic therapy	Anticoagulant strategies after LAAC	Designed to evaluate optimal antithrombotic therapy after LAAC.	Addresses one of the most important unresolved areas in precision LAAC: individualized post-procedural therapy [20]
LALALAND pilot study	Prospective pilot study	Patients with AF undergoing cryoballoon-based LAA isolation and closure	Cryoballoon LAA isolation with closure strategy	Demonstrated feasibility but identified important thrombus concerns after durable LAA isolation	Supports caution when combining electrical LAA isolation with mechanical closure and reinforces the need for surveillance [21]

Abbreviations: AF, atrial fibrillation; DOAC, direct oral anticoagulant; LAAC, left atrial appendage closure; LAA, left atrial appendage; OAC, oral anticoagulation.

**Table 2:** Structured synthesis of selected meta-analyses of left atrial appendage closure.

Study	Evidence Base	Comparison	Main Findings	Key Limitations/Interpretation
Oliva et al. [23]	Systematic review and network meta-analysis; 7 randomized trials; 73,199 participants	LAAC vs. VKAs and DOACs	No significant difference in stroke/systemic embolism between LAAC and VKA or DOAC therapy; bleeding was lower with LAAC after procedural bleeding was excluded.	Network includes indirect comparisons. The mortality signal was versus VKA and should not be extrapolated as a proven mortality advantage over contemporary DOACs.
Kaisaier et al. [24]	Meta-analysis of 4 randomized trials; 3116 participants	LAAC vs. OAC	Similar stroke/systemic embolism; lower hemorrhagic stroke and non-procedural clinically relevant bleeding with LAAC; pooled mortality estimates favored LAAC.	Only four RCTs with heterogeneous populations and anticoagulant comparators. Mortality findings should not be interpreted as proof of superiority over contemporary DOAC therapy.
Wang et al. [25]	Systematic review and meta-analysis; 15 studies, including 4 RCTs and 11 propensity-matched studies; 17,116 participants	LAAC vs. OAC	Pooled analyses reported lower composite events and mortality with LAAC, while stroke and major bleeding were broadly comparable.	Mixing randomized and observational evidence introduces residual confounding and treatment-selection bias, particularly for mortality estimates.
Naeem et al. [26]	Systematic review and meta-analysis reported in the cited conference abstract	LAAC vs. OAC	Provides an updated pooled comparison of thromboembolic, bleeding, and other clinical outcomes.	Conference-abstract evidence provides limited study-level and methodological detail; interpretation should remain cautious.
Davis et al. [27]	Network meta-analysis of 6 randomized trials	Watchman vs. Amulet, with anticoagulation comparators included in the network	Watchman and Amulet showed broadly similar major efficacy outcomes; differences were mainly procedural or device-specific.	Primarily informs device-to-device comparisons rather than the direct clinical question of LAAC versus contemporary anticoagulation.
Lerman et al. [28]	Network and reconstructed individual-patient-data meta-analysis; 12 studies, including 8 RCTs	LAAC vs. warfarin and standard- or low-dose NOACs	Standard-dose NOACs ranked best for stroke/systemic embolism prevention; LAAC was broadly comparable with NOACs and favorable relative to warfarin for selected outcomes.	Relies partly on indirect network comparisons and reconstructed data. Mortality differences involving warfarin should not imply superiority over DOACs.
Juneja et al. [29]	Systematic review and meta-analysis; 14 observational studies; 683,659 participants; preprint	LAAC vs. DOACs	No significant differences in thromboembolic events, major bleeding, all-cause mortality, or cardiovascular mortality.	Preprint composed of observational evidence with substantial potential for residual confounding and heterogeneity; hypothesis-generating only.

**Table 3:** Contemporary LAAC devices and closure strategies according to anatomy and clinical context.

Device/Strategy	Closure Mechanism	Key Advantages	Main Limitations	Potential Clinical/Anatomical Role	Evidence Maturity
Watchman FLX/FLX Pro [2,3,39,40]	Endocardial single-seal plug	Recapturability, procedural familiarity, broad size range, favorable safety profile	Residual PDL in selected complex anatomies; temporary postprocedural antithrombotic therapy usually required	Patients with adequate landing-zone depth; priority on procedural simplicity and repositionability	Established
Amplatzer Amulet [11,12,41]	Endocardial dual-seal lobe-and-disc system	Broad ostial coverage; useful in shallow, wide, or selected complex appendages	Greater procedural complexity; pericardial effusion risk in some studies	Shallow appendage, wide ostium, or anatomy requiring enhanced ostial coverage	Established
LARIAT [4,5]	Epicardial suture ligation	No permanent intracardiac implant; potential reduction in implant-related thrombosis	Requires pericardial access; technically demanding; risk of pericarditis and effusion	Unsuitable endocardial landing zone or selected failed endocardial closure	Selected use
AtriClip [5]	External surgical/epicardial clipping	Durable external exclusion; no intracardiac implant	Requires surgical or thoracoscopic access	Concomitant cardiac surgery or selected thoracoscopic AF procedures	Established in surgical settings
LAmbre [5]	Endocardial umbrella-and-cover system	Adaptable to shallow, irregular, or multilobar anatomy	Limited large randomized comparative evidence	Selected complex appendage morphologies	Emerging clinical
WaveCrest [5]	Endocardial nitinol frame with membrane	Low-profile and repositionable design	Limited contemporary randomized evidence	Selected anatomies; investigational or geographically limited use	Emerging clinical
CLAAS [38]	Conformable foam-based endocardial seal	Passive anatomical conformity; reduced dependence on radial force	Early feasibility evidence; limited long-term outcomes	Potential role in anatomically variable appendages	Early feasibility
Bioresorbable platforms [3]	Temporary scaffold followed by degradation	Potential avoidance of permanent foreign material	Limited human evidence and uncertain long-term durability	Future investigational use	Experimental
Sierra Aegis (Aegis Medical Innovations Inc., Vancouver, BC, Canada) [42]	Percutaneous epicardial ligation	No intracardiac implant; minimal contrast and anticoagulation requirements in early studies	Very small first-in-human experience	Selected patients with renal impairment, bleeding risk, or unsuitable endocardial anatomy	Early feasibility

**Table 4:** Multimodality imaging across the left atrial appendage closure pathway.

Stage/Modalities	Primary Objectives	Main Strengths	Main Limitations	Findings that May Alter Management
Preprocedural TEE [1,6,53]	Exclude LAA thrombus; assess ostium, depth, lobes, and surrounding structures	Real-time imaging; established clinical experience; no radiation or iodinated contrast	Semi-invasive; sedation or anesthesia may be required; limited three-dimensional anatomical coverage compared with CT	LAA thrombus, unsuitable anatomy, inadequate landing-zone depth
Preprocedural cardiac CT/CCTA [1,6,52]	Three-dimensional anatomical characterization, device sizing, landing-zone assessment, procedural-angle planning	High spatial resolution; reproducible measurements; virtual implantation and device-planning capability	Radiation and iodinated contrast; equivocal filling defects may require delayed imaging or TEE confirmation	Device-size adjustment, change in device strategy, identification of complex or unsuitable anatomy
Intraprocedural TEE [1,53,55,56,57]	Guide transseptal puncture, device positioning, compression, stability testing, and immediate leak assessment	High-resolution real-time guidance; established procedural standard	Frequently requires general anesthesia; risk of esophageal injury	Repositioning, resizing, device retrieval, or confirmation of acceptable sealing
Intraprocedural ICE [55,56,57]	Guide transseptal access, deployment, positioning, and leak assessment under local anesthesia	Avoids esophageal instrumentation; supports minimalist workflows	Additional venous access; catheter manipulation; learning curve; potential pericardial or vascular complications	Device repositioning, assessment of stability, immediate complication recognition
Postprocedural TEE [1,47,53]	Detect PDL, DRT, device migration, and incomplete sealing	Widely available; dynamic flow assessment; established follow-up modality	Semi-invasive; may detect fewer small residual channels than CT	Continuation or intensification of antithrombotic therapy; closer follow-up
Postprocedural CT [6,47,53,58]	Evaluate residual LAA patency, PDL anatomy, device position, and endothelialization	Superior spatial resolution; detailed characterization of leak mechanism and residual contrast filling	Radiation, contrast exposure, and possible device-related artifacts	Extended therapy, intensified surveillance, or consideration of repeat intervention
Photon-counting CT [60]	Characterize small leaks and device–tissue interfaces with reduced metal artifact	Ultra-high spatial resolution and improved material discrimination	Limited availability; early clinical evidence	Better definition of fabric deficiency, off-axis deployment, or low-volume residual flow
AI-assisted 3D modeling [59]	Automated segmentation, sizing, and angle prediction	Potentially improves standardization, planning efficiency, and first-pass device selection	Requires external validation, technical integration, and proof of outcome benefit	Device-size prediction, optimal deployment angle, reduced resizing or repeated deployment

## Data Availability

No new data were created or analyzed in this study. Data sharing is not applicable to this article.

## Abbreviations

The following abbreviations are used in this manuscript:

AF	Atrial fibrillation
AI	Artificial intelligence
CCTA	Cardiac computed tomography angiography
CLAAS	Conformal Left Atrial Appendage Seal
CT	Computed tomography
DOAC	Direct oral anticoagulant
DRT	Device-related thrombus
ECG	Electrocardiography
ICE	Intracardiac echocardiography
LAA	Left atrial appendage
LAAC	Left atrial appendage closure
OAC	Oral anticoagulation
PCCT	Photon-counting computed tomography
PDL	Peri-device leak
PET	Polyethylene terephthalate
PFA	Pulsed field ablation
TEE	Transesophageal echocardiography
TT-MAZE	Total thoracoscopic maze
VKA	Vitamin K antagonist
